# Does market segmentation necessarily discourage energy efficiency?

**DOI:** 10.1371/journal.pone.0233061

**Published:** 2020-05-21

**Authors:** Yanjun Yang, Rui Xue, Dong Yang

**Affiliations:** 1 School of Economics and Business Administration, Chongqing University, Chongqing, China; 2 Macquarie Business School, Macquarie University, Macquarie Park, Austrialia; The Bucharest University of Economic Studies, ROMANIA

## Abstract

Prior research tends to propose and examine the negative relationship between market segmentation and energy efficiency. Does market segmentation necessarily impair energy efficiency? Considering the critical role that Chinaese government play in managing erergy efficiency, we propose a non-linear relationship between market segmentation and energy efficiency. Using data of 30 provinces in Mainland China during 2000 to 2017, we find an inverse U-shaped relationship between market segmentation and energy efficiency. Our findings remain robust after controlling endogeneity issues. Therefore, a moderate level of market segmentation is acceptable and beneficial for long-term improvement of energy efficiency in emerging economies.

## Introduction

With the rapid economic growth, the aggregate energy consumption of China has increased dramatically during last decades [1, 2]. In 2018, China remains the largest energy consumer worldwide, accounting for 23.6% of the global energy consumption [3]. Therefore, China plays an important role in mitigating world energy consumption [4]. Huge energy consumption increases the risk of national energy security. To control energy consumption, Chinese government added energy conservation into the long-term strategic planning. As listed in the 11^th^, 12^th^, and 13^th^ five-year plans, China aims to reduce energy consumption per unit GDP by 20%, 17%, and 15%, respectively. However, it is hard to balance the tradeoff between energy conservation and rapid economic growth [5, 6]. Under this circumstance, improving energy efficiency is widely accpeted as an effective approach to achieve the balance [7, 8, 9].

As the largest transition economy of the world, China has adopted an export-oriented economic development mode. In order to increase local GDP and tax revenue, local governments use their administrative powers on important issues such as trade and credit to implement various forms of protection for local enterprises, especially large state-owned enterprises (SOEs). Specific measures include setting up trade barriers for external enterprises, providing preferential policies and financial support to local enterprises, and preventing the outflow of local production factors and inflow of products from other places, which impedes the formation of market integration [10, 11]. As a result, energy efficiency demonstrates a dysfunctional pattern [7]. Specifically, local governments ignore the limitations of its own resource endowments and prioritise establishing high energy-consuming industries, which not only cause serious production overcapacity and energy waste, but also generate serious environmental pollutions [12]. Wei and Zhang [13] argue that market segmentation has a negative impact on the improvement of energy efficiency, manifest in reducing scale effect, restricting competition and technology diffusion. Nie and Zhang [14] provide empircial evidence that factor market distortion, enterprises R&D investment, and industrial agglomeration are three mediation mechanisms through which market segmentation inhibits energy efficiency. Although prior studies have analysed driving factors of energy efficiency, a consideration for the positive role that local governments play in energy efficiency improvement receives little attention and awaits empirical testing.

The mechanism of how market segmentation restrains energy efficiency is based on the premise that perfect market mechanism (price mechanism) guides resource flow. In a market where the market mechanism works well, the price mechanism can ensure the producers who value production factors most are most likely to obtain the factors, thereby maximizing the the production efficiency; However, perfect price mechanism is not fully applicable in China’s energy market because of the long-term undervaluation of energy price [15]. Therefore, local government, as a supplement to the intangible hand of market, does not necessarily inhibit the improvement of energy efficiency; instead, in some cases, it could improve the energy efficiency. For example, in 2006, the central government increased the weight of energy conservation and emission reduction in the performance assessment system of local officials, and prioritised promotion of officials with outstanding performance in energy conservation and emission reduction work. As a result, local governments were highly incentivated to make contributions to energy conservation and carbon emission reduction [16]. The local governments assist local enterprises in upgrading production technology through financial support and policy preferences [17, 18], In 2010, Chinese local governments’ total financial subsidy to support energy conservation and emission reduction reached CN¥29.7 billion (about US$4.39 billion at 2010 exchange rate). Support for local entreprises is likely to not only improve local energy efficiency, but also shape a healthy competition environment through mutual learning [19].

In addition, within the context of open-up economic development mode, foreign markets have a substitute for domestic markets, local entreprises compete with foreign products and services which requires higher standard for technology and operation of local firms. To guarantee local firms’ survival within the competitive environment, local governments tend to lower credit requirements and support local enterprises to carry out technical transformation and upgrading [20]. With advanced production technology and sound strategic management, local firms are more likely to manufacture and operate in a more energy-friendly way.

Taken together, the impact of market segmentation on energy efficiency is unclear and awaits empirical investigation. We propose an inverse U-shaped relationship between market segmentation and energy efficiency. Specifically, the impact of market segmentation on energy efficiency is positive when market segmentation level is relatively low; the positive impact turns negative when market segmentation exceeds a certain level. Recent research provids some evidence to support our proposition. Jin and Zhao [21] and Sun et al. [22] both find that market segmentation has two-sided effects on factor productivity. On the one hand, market segmentation restrains the improvement of factor productivity by reducing the scale effect of resources, the effectiveness of resources allocation, and technology sharing [23]. On the other hand, market segmentation improves the competitiveness of local enterprises by setting up barriers to external firms, which improves local factor productivity [18]. Specifically, when the level of market segmentation is low, market segmentation wields positive influences on factor productivity and improves overall factor productivity. However, when market segmentation exceeds a certain level, further degree of market segmentation impairs factor productivity [19].

In this paper, we use panel data of 30 provinces from Mainland China during 2000 to 2017 to investigate the effects of market segmentation on energy efficiency. We further implement the generalized method of moments (GMM) of dynamic panel and threshold regression model to check the robustness of the baseline model. The results document an inverse U-shaped relationship between market segmentation and energy efficiency. The robustness checks provide evidence that our results are robust and reliable. Compared to the negative relationship suggested in existing researches, we provide a more nuanced and complete understanding of market segmentation and energy efficiency. The findings are important for policy makers as moderate market segmentation is indeed advantageous for improving energy efficiency. Either too low or too high levels of market segmentation is detrimental for energy efficiency.

This paper makes contributions to exisiting literature in two main fronts. First, different from existing studies [8, 9, 20], we take into account the role that local government plays in resource allocation as a supplement to the market mechanism based on the premise of the characteristics of China’s economic transition. We propose an inverted U-shaped relationship between market segmentation and energy efficiency. Our analysis shows that in emerging market like China, market segmentation is not expected as low as possible; in effect, moderate market segmentation is conducive to the improvement of energy efficiency. Second, in contrast to previous studies using data envelopment analysis (DEA) method or traditional stochastic frontier analysis (SFA) method, this study applies the newly developed SFA approach to calculate energy efficiency. It takes into account large differences and heterogeneities between provinces of China. Therefore, it can not only capture the unobserved heterogeneity, but also reduce measurement errors of energy efficiency by separating invalid elements from data error and statistical noise.

The rest of this paper is structured as follows. Section 2 reviews prior research and summarises research gaps. Section 3 describes the data, variables, and introduces the econometric model. The baseline results and robustness checkes are implemented in section 4. Section 5 concludes and proposes policy recommendations.

## Literature review

Research on China’s energy efficiency includes two strands. One strand of the literature focuses on the measures of energy efficiency. Patterson (1996) is the seminal research contributor in energy efficiency measurement area which used energy intensity (the ratio of energy consumption to GDP) to measure energy efficiency [24]. However, Nagata (1997) argues that energy intensity is not a good measure of energy efficiency as it is largely affected by economic structure [25]. Hu and Wang (2006) propose the concept of total factor energy efficiency (TFEE) within the framework of total factor productivity, and utilise data envelopment analysis (DEA) method to measure the TFEE [26]. Specifically, TFEE is a ratio of target energy input to actual energy input. Subsequent research builds on and expands DEA models, and develops various DEA variants, taking into account differenct components of energy efficiency. These models are all non-parametric method as no specific function form is specified. Compared to non-parametirc models such as DEA and its variants, the stochastic frontier analysis (SFA) model is a parametric model with specifications of efficiency function, which outperforms non-parametric models in some cases but also increases measurement errors due to inappropriate model specifications. Compared to traditional SFA, the recently developed parametric SFA model, proposed by Kumbhakar et al. [27], improves measurement accuracy by specifing different errors which could capture information on individual heterogeneity, time-variant inefficiency, time-invarient inefficiency, and random shocks, respectively [28, 29].

The second strand of existing research concentrates on identifing driving factors of energy efficiency at national, regional or industrial level [7, 12, 30, 31, 32, 33, 34]. However, piror researches emphasise the impact of economic factors on energy efficiency, and fail to take into account the impact of market segmentation on energy efficiency [8, 9]. In China, market segmentation has a strong impact on the economy because local governments dominate local economic development mode, policies and regulations [8]. In China. political connections and embeddedness play an important role in economic development and affect firms’ credit support and resource distribution [35]. To protect local firms, local governments control the production of raw materials and set up barriors to purchase goods produced by other provinces, which results in serious market segmentation [12, 13]. Market segmentation has been considered as the “stumbling block” for China’s long-term sustainable economic development [36]. This is because it affects the efficiency of resource utilisation through the following two ways. Firstly, market segmentation reduces the scale effect of resource allocations [12]. In a segment market, the allocation of resources determined by price mechanism only exists in small markets but not in large markets. As a result, effective energy consumers are not allocated with enough resource supplies whereas inefficient consumers obtain surplus resources, resulting in a decline in the effectiveness of overall resource use [37]. Moreover, local governments tend to protect local enterprises through administrative intervention to prevent more competitive external enterprises from entering the local market, which is clearly detrimental for the efficiency of resource allocation [38]. The efficiency of resource is also negatively affected by the limited spillover effects of inactive interregioanl cooperations [39, 40]. Secondly, market segmentation reduces technology advancement. In the segment market, compared to foreign and external entreprises, local enterprises, especially local state-owned entreprises, are more likely to obtain funding at lower costs through potilical connections, and so they have less motivations to invest in technology research and development [41]. This significantly and negatively affects technology development [42, 43]. Through the above two ways, energy utilisation is thus likely to be affected by market segmentation because market segmentation has a negativeeffect on scale effect and technology diffusion [13] In addition, Nie and Zhang [14] find that market segmentation has a significant and negative impact on energy efficiency, but such negative impact disappears in regions with low levels of market segmentation.

To sum up, from different perspectives, prior literatures tend to support a negative relationship between market segmentation and energy efficiency [8, 9, 13, 14, 19, 35]. Moreover, various DEA models and traditional SFA models are utilised in prior researches to measure energy efficiency; however, these models are not likely to split individual heterogeneity, time-varying, and time-invarient chareteristics, as well as random shocks.

In this paper, the newly developed SFA method is used to measure provincial energy efficiency in China, which takes into account more information and improves measurement accuracy. More importantly, building on the two opposite views on the influences of market segmentation on factor production [8, 9], we propose an inverse U-shaped relationship between market segmentation and energy efficiency. Considering the critical role that China local governments plays in managing energy consumption, we illustrate a more nuanced and complete picture concerning the non-linear relationship between market segmentation and energy efficiency.

## Method

### Data

This study collects sample data from 30 provinces in Mainland China from 2000 to 2017. Tibet was not incluced due to data availability. Key variable and control variables are collected from China Energy Statistical Yearbook, China Statistical Yearbook, China Statistical Yearbook of Environment, National Bureau of Statistics, and National Intellectual Property Administration. We replace missing data through interpolating with time-series mean value.

### Variables

#### Measurement of energy efficiency

Mainstream methods utilised of measuring energy efficiency include non-parametric and parametric models. Non-parametric methods, such as DEA and its variants, have no explicit function form and are widely used in the case of small sample size. Parameter methods, such as SFA and its varients, manifest in a consideration for unobservable heterogeneity by setting a specific form of frontier function. In other words, compared to non-parametric method, the main advantage of parametric method is to separate invalid components from statistical noises caused by data error and missing information. This is important as individual heterogeneity is the main part for panel data and the core of statistical analysis. Among SFA varients, the recently developed SFA model by Kumbhakar et al. [24] spilts errors into four fronts, enabling the estimates to capture information on individual heterogeneity, time-variant inefficiency, time-invarient inefficiency, and random shocks, respectively [25, 26]. Therefore, due to the specific national condition and economic transition mode of China, we apply Kumbhakar et al.’s [24] SFA model to measure provincial energy efficiency.

Following Kumbhakar et al. [24] and Filippini and Hunt [25], we construct the following model to measure energy efficiency:

$${\text{e}}_{it}=\alpha_{0}+\beta^{\prime }x_{it}+\mu_{i}+\nu_{it}-\eta_{i}+u_{it}$$ (1)

where e_*it*_ is the natural logarithm of aggregate energy consumption of province *i* at year *t*. *x*_*it*_ is a set of inputs and outputs covariates. Following Filippini and Hunt [25], we include the natural logarithm of real GDP, the natural logarithm of total population, the natural logarithm of population density, the natural logarithm of constant energy price index, the share of added value of industrial sector to GDP. All of these variables are price deflated by 2000 price level. The error term is split into four components. Specifically, *u*_*it*_ and *η*_*i*_ are time-varying and time-invarient inefficiency; *μ*_*i*_ and *v*_*it*_ are province fixed effect and noise, respectively. As mentioned before, these four factors cannot be captured by other energy efficiency models at the same time. Therefore, the use of Kumbhakar et al.’s [24] measure includes more information and improves the measurement accuracy.

Estimation of the formula (1) is implemented using a single stage maximum likelihood (ML) method basedon distributional assumptions on the four components [44]. To facilitate the estimation, the model can be rewritten as follows:

$${\text{e}}_{it}=\alpha_{0}^{*}+\beta^{\prime }x_{it}+\alpha_{i}+\varepsilon_{it}$$ (2)

Where $\alpha_{0}^{*}=\alpha_{0}-E(\eta_{i})-E(\mu_{it})$; *α*_*i*_ = *μ*_*i*_ − *η*_*i*_ + *E*(*η*_*i*_); *ε*_*it*_ = *v*_*it*_ − *u*_*it*_ + *E*(*u*_*it*_). With this specification *α*_*i*_ and *ε*_*it*_ have zero mean and constant variance, Specifically, there are three steps to estimate the model.

Step 1: We apply panel data model to obtain estimate $\hat{\beta^{\prime }}$, and make predictions of *α*_*i*_ and *ε*_*it*_, denoted as ${\hat{\alpha }}_{i}$ and ${\hat{\varepsilon }}_{it}$.
Step 2: Estimate the time-varying inefficiency *u*_*it*_.
  $$\varepsilon_{it}=\nu_{it}-u_{it}+E(u_{it})$$ (3)
  Where *ε*_*it*_ is the predicted value obtained in step 1, *v*_*it*_ follows $N(0,\sigma_{\nu }^{2})$, and *u*_*it*_ follows $N^{+}(0,\sigma_{u}^{2})$. We can estimate Eq (3) using standard SFA technique to get the prediction of the time-varying inefficiency ${\hat{u}}_{it}$. Using the predicted time-vary inefficiency, we calculated the residual of the time-varying inefficiency (RTE), $RTE=\text{exp}(-{\hat{\mu }}_{it})$.
Step 3: Estimate *η*_*i*_ using a similar process with step 2. We replace *α*_*i*_ in the following equation with ${\hat{\alpha }}_{i}$ obtained from step 1.
  $$\alpha_{i}=\mu_{i}-\eta_{i}+E(\eta_{i})$$ (4)

Similarly, *μ*_*i*_ and *η*_*i*_ follow $N(0,\sigma_{\mu }^{2})$ and $N(0,\sigma_{\eta }^{2})$, respectively. Applying Jondrow et al.’s [45] method to Eq (4), we can obtain the prediction of persistent time-invarient inefficiency (PTE), which is $PTE=\text{exp}(-{\hat{\eta }}_{i})$. Lastly, energy efficiency *(EE*) is obtained by the product of PTE and RTE, namely *EE* = PTE*RTE.

**Fig 1:**
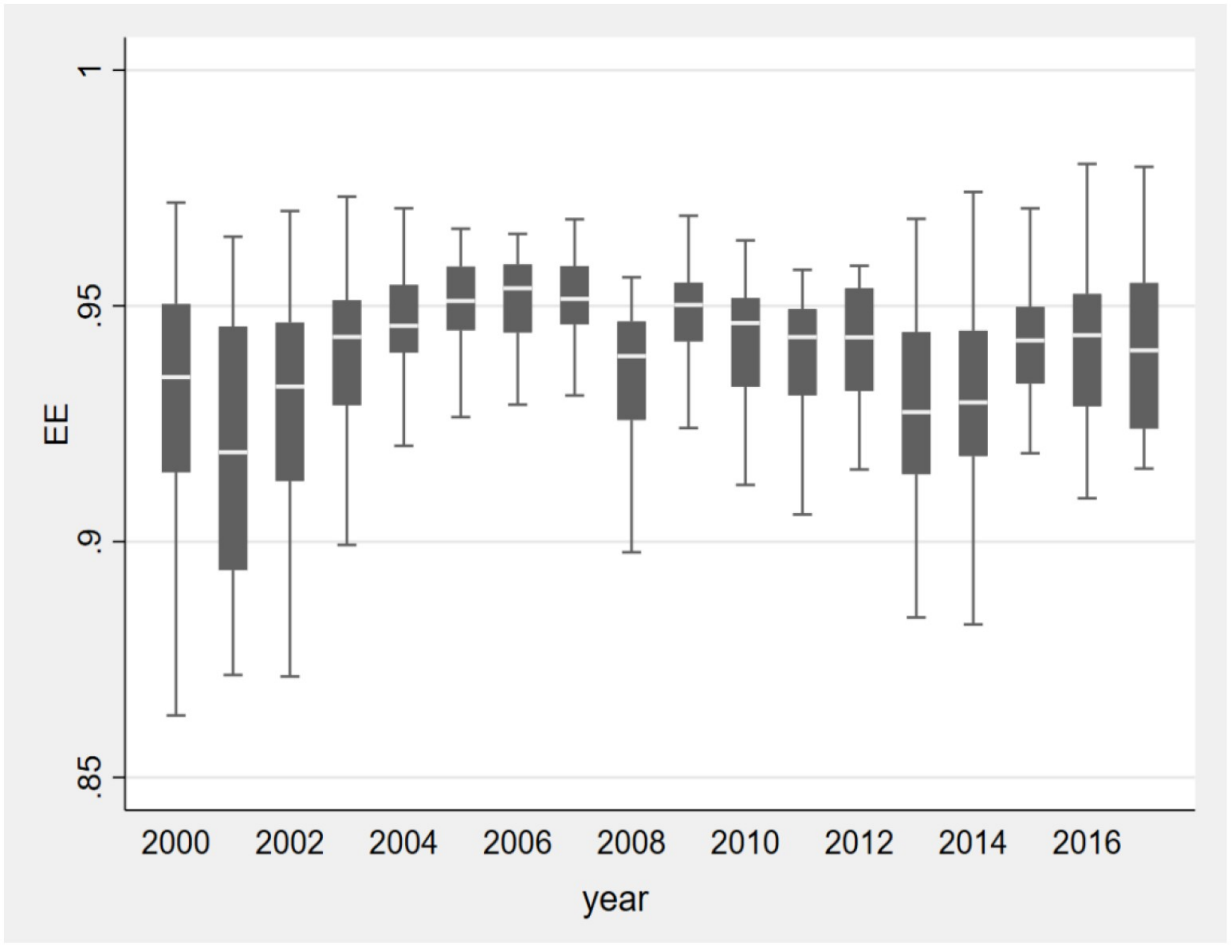
Average energy efficiency in China from 2000 to 2017.

The results of estimated average *EE* are illustrated in Fig 1 with two-year intervals. As shown in the Fig 1, China’s energy efficiency fluctuates during the last two decades. It demonstrates a up-down-up pattern. Overall, there is an increase from 2000 to 2017. It is notable that the global financial crisis (GFC) in 2008 has a considerable adverse impact on China’s energy efficiency. Before GFC, China’s energy efficiency shows a steady upward trend, while it begins to increase until 2015 following GFC, which is consistent with Zhu et al. [31]. In addition, there is a obvious decline in 2013 and 2014. A possible explanation is the decline in economic growth at the end of 2012. To stabilise economic, local governments have relaxed regulations of energy-intensive companies.

#### Measurement of market segmentation

A number of methods have been used to measure market segmentation, such as "Production Law" [46] and "Trade Law" [10]; however, these methods have certain shortcomings on information loss that cannot measure market segmentation well [16, 47]. Another strand of studies use commodity price to evaluate market segmentation. It is build on Glacier Cost Model [48] and further developed by Parsley and Wei [49]. They argue that the degree of market segmentation is increasing when the price difference between regions increases, and vice versa. Recent studies utilise this method to investigate market segmentation in China, such as Li and Lin [8], and Wei and Zheng [9].

Following Li and Lin [8], we choose the retail price indices of food, beverages, clothing & shoes, commodity, medical & healthcare supplies, books & magazines, fuels, and building materials & hardware to construct the market segmentation (MS) index. The MS index can be constructed as follows.

First, we define $p_{it}^{k}$ as the price of product *k* in province *i* at year *t*, *k* ∈ {*k*_1_, *k*_2_, ⋯, *k*_8_}; so the price difference between province *i* and *j* for a given product *k* at year *t* is:

$$\Delta Q_{ijt}^{k}=\text{ln}\left(\frac{p_{i,t}^{k}}{p_{j,t}^{k}}\right)-\text{ln}\left(\frac{p_{i,t-1}^{k}}{p_{j,t-1}^{k}}\right)=\text{ln}\left(\frac{p_{i,t}^{k}}{p_{i,t-1}^{k}}\right)-\text{ln}\left(\frac{p_{j,t}^{k}}{p_{j,t-1}^{k}}\right)$$ (5)

Next, as the $\Delta Q_{ijt}^{k}$ suffers from the measurement bias from heterogeneity of products, which is irrelevant to market segmentation, we have to exclude the non-additive effect caused by the heterogeneity in $\Delta Q_{ijt}^{k}$. Following Parsley and Wei [47] and Qin et al. [35], we adopts the demean method to control for cross-sectional dependencies and substracts the mean price differnce from the $\Delta Q_{ijt}^{k}$. Hence, the relative price change is re-calculated as below:

$$q_{ijt}^{k}=\left|\Delta Q_{ijt}^{k}\right|-\left|\bar{\Delta Q_{t}^{k}}\right|$$ (6)

Lastly, we aggregate the relative price change of all eight products and calculate its variance of 66 pairwise combinations of adjacent provincial units. Therefore, the MS index can be obtained using following equations:

$$V\text{ar(}q_{ijt})=\text{Var}(q_{ijt}^{k_{1}},q_{ijt}^{k_{\text{2}}},q_{ijt}^{k_{\text{3}}}\text{,}q_{ijt}^{k_{\text{4}}},q_{ijt}^{k_{\text{5}}},q_{ijt}^{k_{{}_{\text{6}}}}\text{,}q_{ijt}^{k_{{}_{\text{7}}}},q_{ijt}^{k_{\text{8}}}\text{)}$$ (7)

segit=∑i≠jVar(qijt)N (8)

where Var(*q*_*ijt*_) represents the price disperse which is the variance of aggregate price changes between province *i* and *j* at year *t*; *seg*_*it*_ denotes the MS index of province *i* at year *t*, *N* is the number of pairs of adjacent provinces. Compared to prior research including one or only a few products, the MS index constructed in this paper contains more types of products, which can reflect more information concerning the segmentation of the whole market. Additionally, to avoid a large or small regression coefficient and make interpretations more straightforward, we enlarge the value of MS by 100 times.

**Fig 2:**
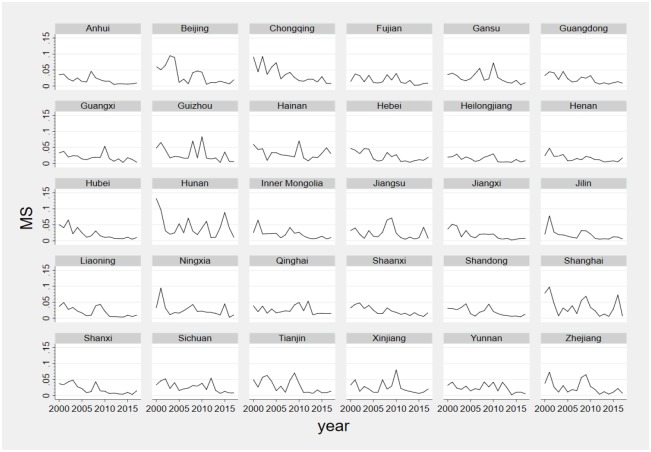
MS index of 30 individual provinces in China.

Fig 2 illustrates the MS index of individual provinces in China. As illustrated in the Fig 2, the MS index of most provinces shows a downward pattern, which is a likely result from the promotion of China’s market-oriented reform and regional economic integration policy. Besides, during 2008 and 2010, market segmentation in most provinces increased, which is consistent with the findings from Wei and Zheng [9] and Qin et al. [35]. In addition, some provinces experienced considerable fluctuations in market segmentation, (e.g., Hunan, Beijing, Shanghai,), while other provinces remain unchanged or changed in a limited range, (e.g. Heilongjiang, Henan, Anhui).

#### Control variables

We include a number of variables to control for their respective effects on energy efficiency. *Openness* (OPE), according to Pan et al. [50], foreign direct investment (FDI) has narrowed the gap between capital and foreign exchange, bring about advanced management approaches and technology, which has an important impact on the improvement of China’s energy efficiency. We use the ratio of FDI to GDP to measure Openness. *Energy price* (PI), energy price is an important external factor influencing the energy efficiency in China [7]. Higher energy price increases costs on firms’ operation budget, which further affects the energy efficiency. We use the energy price index to reflect changes in energy prices. *Government environmental regulation* (GOV), in China, local governments have a direct and considerable impact on the corporate production activities. Given the fact that environmental regulations issued by local governments affect energy efficiency through industrial structure and technological progress, we use the ratio of investment in pollution control to GDP to measure government environmental regulation consistent with Nie and Zhang [14]. *Industry structure* (INDU), industry structure is a reflection of productivity, which determines the quality of energy consumption. We use the share of added value of industrial sector to GDP to measure industry structure. *Economic development* (ES), economic development is highly correlated with energy efficiency. We use real GDP per capita to measure the economic development. *Technology advancement* (TEC), technology plays a dominant role in improving energy efficiency [34, 51]. We apply the number of patents granted to represent technology advancement. Table 1 describes key and control variables and their measures.

**Table 1:** Descriptive statistics of variables.

	Explanations	Obs	Mean	Sd.	Min.	Max.
**EE**	energy efficiency index	540	0.9381	0.0218	0.8561	0.9801
**MS**	market segmentation index	540	0.0243	0.0191	0.0022	0.1318
**OPE**	FDI/GDP	540	0.4033	0.3828	0.0607	1.8654
**TEC**	number of patents granted	540	22295	44565	70	332652
**GOV**	investment in environmental pollution treatment/GDP	540	0.0130	0.0064	0.0030	0.0423
**INDU**	the proportion of industrial added value in GDP	540	0.3871	0.0846	0.1184	0.5924
**ES**	real GDP per capita	540	10997	7286	2759	42833
**PI**	price index for fuels	540	1.3773	0.3932	0.8387	2.6092

This table displays the number of observations, the mean, standard deviation, minimum and maximum values of the dependent, explanatory and control variables.

### Model specification

We examine the relationship between market segmentation and energy efficiency by estimating the following regression modelling method:

$$\text{ln}EE_{it}=\gamma_{0}+\gamma_{1}\text{ln}MS+\gamma_{2}{\left(\text{ln}MS\right)}^{2}+\gamma_{\text{3}}X_{it}\text{+}\lambda_{t}+\mu_{i}+\varepsilon_{it}$$ (9)

Where ln*EE*_*it*_ stands for the natural logarithm of energy efficiency of province *i* at year *t*; ln*MS* represents the natural logarithm of market segmentation; *X* is the control variables matrix described above, including *Openness*, *Energy price*, *Government environmental regulation*, *Economic development*, *Industry structure*, and *Technology advancement*. *λ*_*t*_ is the fixed effect used to control the heterogeneity over time and *μ*_*i*_ is the fixed effect used to control the province heterogeneity. *ε*_*it*_ is the random error term, *γ*_0_ − *γ*_3_ are the parameters to be estimated.

## Results

### Scatterplot

**Fig 3:**
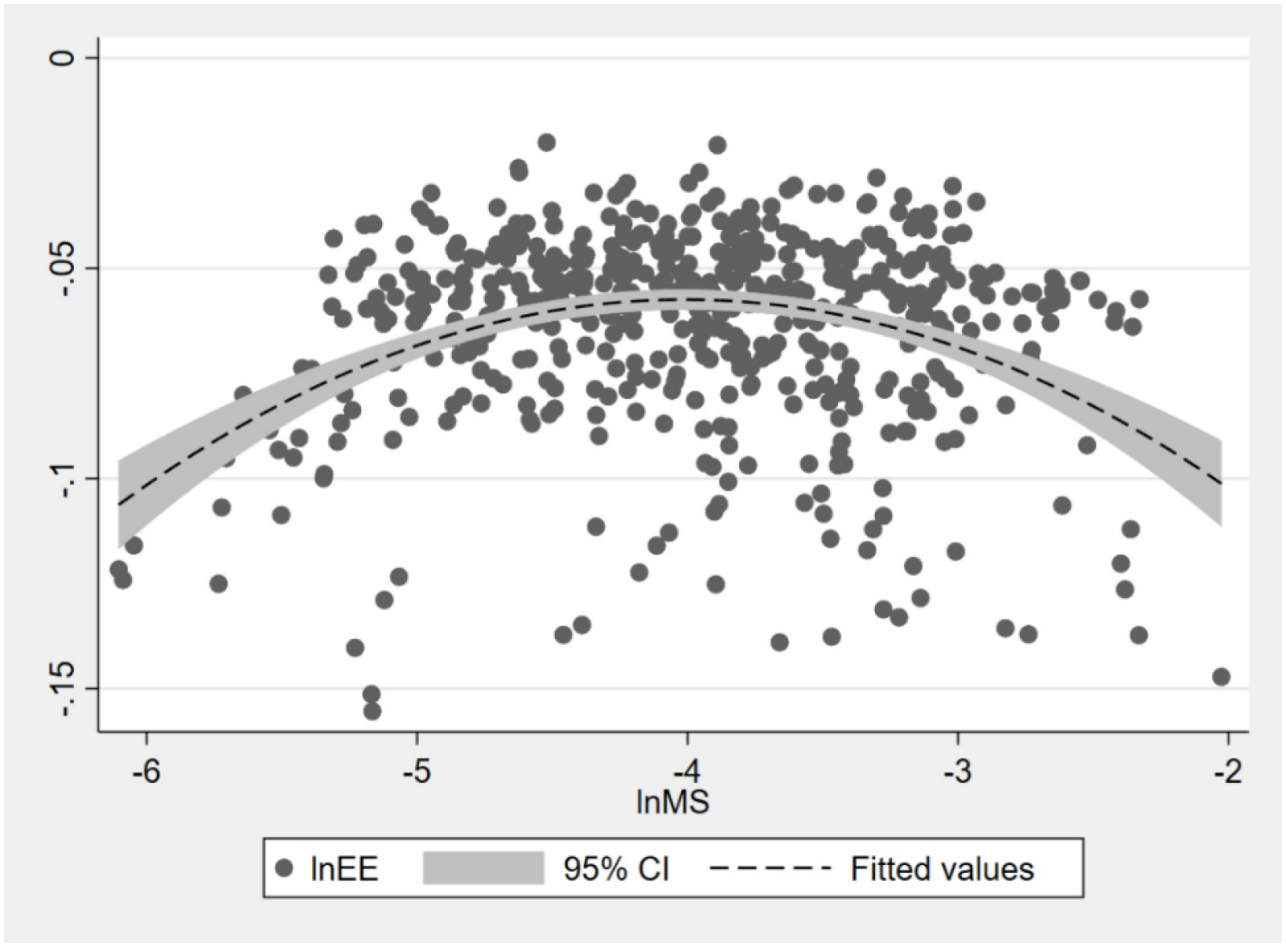
Scatterplot of market segmentation and energy efficiency.

Fig 3 illustrates the scatterplot of market segmentation and energy efficiency. It provides preliminary information of the segmentation-efficiency nexus. As shown in Fig 3, there is an inverse U-shaped relationship between market segmentation and energy efficiency. Whether this non-linear relationship is statistically significant is examined by the regression modelling and the robustness checks.

### Panel unit root and cointegartion test

Since panel data is likely to fall into the pseudo-regression trap caused by serious time trends, the unit root needs to be checked before implementing regression modelling. To make the results more reliable, this study uses four methods to test the unit root, including LLC test, HT test, ADF-fisher test, and IPS test. The results are shown in Table 2.

**Table 2:** Unit root test results of panels.

	LLC	HT	ADF-fisher	IPS
***lnEE***	-5.6599***	-6.4033***	151.5877***	-6.1762***
	(0.0000)	(0.0000)	(0.0000)	(0.0000)
***lnMS***	-6.6313***	-16.2709***	408.1806***	-10.7558***
	(0.0000)	(0.0000)	(0.0000)	(0.0000)
***lnOPE***	-1.5499*	-6.3370***	76.7597*	-0.9350
	(0.0606)	(0.0000)	(0.0712)	(0.1749)
***lnTEC***	-3.3143***	-1.2548	73.1033	-3.4147***
	(0.0005)	(0.1048)	(0.1193)	(0.0003)
***lnGOV***	-3.7316***	-8.9396	129.2676***	-2.8075***
	(0.0001)	(0.0000)	(0.0000)	(0.0025)
***INDU***	-2.2847**	1.3796	49.9897	1.4034
	(0.0112)	(0.9161)	(0.8182)	(0.9197)
***lnES***	0.6783	-5.0402	136.8998***	-0.1694
	(0.7512)	(0.0000)	(0.0000)	(0.4327)
***lnPI***	-3.8660***	0.8301	67.9674	-0.8091
	(0.0001)	(0.7968)	(0.2243)	(0.2092)
***ΔlnEE***	-9.9407***	-37.1747***	755.2857***	-12.1703***
	(0.0000)	(0.0000)	(0.0000)	(0.0000)
**Δ*lnMS***	-14.3723***	-41.7153***	666.6231***	-13.7654***
	(0.0000)	(0.0000)	(0.0000)	(0.0000)
***ΔlnOPE***	-2.5950**	-32.2589***	350.6515***	-7.8233***
	(0.0047)	(0.0000)	(0.0000)	(0.0000)
***ΔlnTEC***	-6.1626***	-29.3598***	406.3076***	-9.2731***
	(0.0000)	(0.0000)	(0.0000)	(0.0000)
***ΔlnGOV***	-10.1322***	-37.4027***	456.4164***	-11.3897***
	(0.0000)	(0.0000)	(0.0000)	(0.0000)
***ΔINDU***	-2.6858***	-22.1003***	224.7546***	-7.7760***
	(0.0036)	(0.0000)	(0.0000)	(0.0000)
***ΔlnES***	-3.8796***	-29.2907***	375.0246******	-9.8171***
	(0.0001)	(0.0000)	(0.0000)	(0.0000)
***ΔlnPI***	-7.1549***	-25.9680***	362.0321***	-9.5942***
	(0.0000)	(0.0000)	(0.0000)	(0.0000)
***results***	I(1)	I(1)	I(1)	I(1)

***Δ*** is a first-order differential label, the “demean” option is added to the panel unit root test to alleviate the possible autocorrelation problems, and the lag period is selected according to AIC. Among them, the original sequence test takes the trend item and the difference test is without trend item. In parentheses are P-value of the statistic.

*** p<0.01,

** p<0.05,

* p<0.1.

As can be seen from Table 2, the P-values of some horizontal variables in the four methods are greater than the 10% significance level, suggesting the existance of a unit root. Therefore, we continue to test the first-order difference of the variables. The results show that all the first-order difference variables are stationary at the 1% level.

The unit root tests show that both the dependent variable and independent variable satisfy the conditions of first-order cointegration. Therefore, we apply the panel cointegration test proposed by Pedroni [52] to test the cointegration relationship. The results of the cointegration test are reported in Table 3. It is clear that all statistics are significant at the 1% significance level, which reject the null hypothesis that there is no cointegration relationship. In other words, all variables included in the regression modelling have a long-term cointegration relationship. Therefore, we can apply these variables directly to the regression modelling.

**Table 3:** Results of panel cointegration test.

	Statistic	P-value
***Modified Phillips-Perron t***	7.6795	0.0000
***Phillips-Perron t***	-14.2307	0.0000
***Augmented Dickey-Fuller t***	-14.0352	0.0000

The original assumption is that the panel has no cointegration, alternative hypothesis is all panels are cointegrated.

### Baseline results

Table 4 shows the baseline results of the regression modelling. Model 1 is the fixed effect model that excludes the quadratic term of market segmentation, It shows that the coefficient of the market segmentation is positive significant, which contradicts the negative relationship examined in some prior researches [13, 14]. There may be two reasons for this contradiction. Firstly, different from existing studies which used non-parametric estimates of energy efficiency, the SFA method used in this paper takes into account the heterogeneity between different regions (provinces). In this way, we can not only capture unobserved region-specific characteristics, but also separation the errors and statistical noise from data [28]. Secondly, endogenous bias may exist using fixed effect models. This is reflected in our robustness checks when we control the endogeneity (in Table 5) that the coefficient of market segmentation is significant and negative. Model 2 shows the results of a fixed-effect model with a quadratic term for market segmentation included. Hausman test (Hausman = 21.3800, P-value = 0.0032<0.01) shows that it is reasonable to choose the fixed effect model. In addition, the BP test (P-value = 0.0071<0.01) shows that the model suffers a problem of heteroscedasticity. Therefore, Models 2 to 4 estimate the regression models with an inclusion of robust variance. Model 3 and Model 4 take into account time fixed effect, and both province and time fixed effects, respectively. As the adjusted R^2^ is highest in Model 4 and the likelihood ratio test (LR = 89.45, P = 0.0000) shows the superiority of this mdel, we interpret our results based on Model 4. The results show that the coefficients of *lnMS* and (*lnMS*)^2^ are -0.0727 (p<0.001) and -0.0099 (p<0.001), respectively. Consistent results are also found in Model 2 and Model 3. Therefore, these findings support our proposition that there is an inverse U-shaped relationship between market segmentation and energy efficiency. We also report the turning point of the inverse U-shaped curve in the last row of Table 4. It shows that the turning point is -3.6717, which indicates that energy efficiency improves as market segmentation increases before MS index reaches -3.6717 and decreases when MS index is greater than -3.6717.

**Table 4:** Baseline results.

	Model 1	Model 2	Model 3	Model 4
***lnMS***	0.0042**	-0.0880***	-0.0760***	-0.0727***
	(0.0017)	(0.0107)	(0.0125)	(0.0122)
***(lnMS)***^***2***^		-0.0114***	-0.0101***	-0.0099***
		(0.0014)	(0.0016)	(0.0016)
***lnOPE***	0.0147***	0.0103*	0.0035*	0.0083
	(0.0037)	(0.0045)	(0.0017)	(0.0089)
***lnTEC***	-0.0015	0.0012	0.0043***	0.0073
	(0.0022)	(0.0038)	(0.0014)	(0.0083)
***lnGOV***	0.0066*	0.0073*	0.0077*	0.0101*
	(0.0034)	(0.0036)	(0.0043)	(0.0049)
***INDU***	0.0158	0.0008	-0.0173	-0.0687
	(0.0240)	(0.0375)	(0.0184)	(0.0581)
***lnES***	0.0227*	0.0185	-0.0017	0.0151
	(0.0116)	(0.0162)	(0.0040)	(0.0328)
***lnPI***	0.0178**	0.0050	-0.0115	-0.0160
	(0.0083)	(0.0162)	(0.0083)	(0.0211)
***Intercept***	-0.2057**	-0.3620*	-0.1840***	-0.3110
	(0.0928)	(0.1767)	(0.0507)	(0.2876)
***Hausman test***		21.3800		
		[0.0032]		
***AdjR***^***2***^	0.0640	0.1654	0.2571	0.5680
***N***	540	540	540	540
***Turning point***		-3.8596	-3.7624	-3.6717

*** p<0.01,

** p<0.05,

* p<0.1;

Model 1 is the panel data model with province fixed effects excluding (lnMS)^2^;

Model 2 is the baseline panel data model with province fixed effects;

Model 3 is the panel data model with time fixed effects;

Model 4 is the panel data model with both province and time fixed effects.

In square bracket is the P-value of Hasuman test;

In parentheses are robust standard error.

**Table 5:** Dynamic panel regression results.

	Diff-GMM		Sys-GMM	
	Model 1	Model 2	Model 3	Model 4
***l*.*lnEE***	0.3778***	0.3321**	0.6232***	0.5296**
	(0.0272)	(0.0540)	(0.0529)	(0.0684)
***lnMS***	-0.0013*	-0.0850***	-0.0020**	-0.1193***
	(0.0008)	(0.0088)	(0.0008) **	(0.0123)
***(lnMS)***^***2***^		-0.0105***		-0.0146***
		(0.0011)		(0.0015)
***lnOPE***	0.0344***	0.0309**	0.0084**	0.0084**
	(0.0049)	(1.0073)	(0.0039)	(0.0034)
***lnTEC***	-0.0036	-0.0011	0.0004	0.0006
	(0.0024)	(0.0016)	(0.0021)	(0.0010)
***lnGOV***	0.0042**	0.0084	0.0121***	0.0144*
	(0.0020)	(0.0045)	(0.0046)	(0.0048)
***INDU***	0.0582**	0.0453	0.0302	-0.0014
	(0.0256)	(0.0286)	(0.0365)	(0.0215)
***lnES***	-0.0246**	0.0149	-0.0106	-0.0019
	(0.0117)	(0.0109)	(0.0089)	(0.0060)
***lnPI***	0.0320***	0.0344	0.0024	0.0007
	(0.0081)	(0.0077)	(0.0095)	(0.0139)
***Intercept***			0.1135*	-0.1779
			(0.0621)	(0.0691)
***Ar(1) test***	0.0000	0.0000	0.0000	0.0000
***Ar(2) test***	0.8310	0.2020	0.6220	0.2520
***Hansen test***	1.0000	1.0000	1.0000	1.0000
***Turning point***		-4.476		-4.0856
***N***	480	480	510	510

*** p<0.01,

** p<0.05,

* p<0.1.

In addition, the coefficient of *lnGOV* positive and statistically significant at 10% significance level, indicating that government environmental regulation plays a significant and positive role in improving energy efficiency with coefficient of 0.0101 (p<0.1000) [48]. the coefficient of *INDU* is not significant at conventional levels. Consistent to Wang et al. (2019), the impacts of ln *ES* and ln *PI* on energy efficiency are not observed in China [7].

### Robustness checks

#### Endogeneity issues

Our empirical analysis may suffer from endogeneity problems. This may result from two factors. First, energy efficiency is likely to be affected by past factors as improving energy efficiency is a dynamic process in the long run. A lack of consideration for past factors may lead to estimation bias. Second, energy efficiency could have a feedback effect on market segmentation because provinces with low energy efficiency are likely to receive lower returns and so they are more likely to protect local firms and set financial and political barriers for external firms, which in turn results in heavier market segmentation. Therefore, to mitigate endogeneity problems, we implemented dynamic panel Generalised Method of Moments (GMM) [53]. Both difference GMM (Diff-GMM) and system GMM (Sys-GMM) are utilised for robustness checks.

Table 5 reports the results of Diff-GMM and Sys-GMM, respectively. As shown at the bottom of Table 5, Arellano-Bond Test for AR(1) and AR(2) displays a first-order sequence correlation and a second-order sequence uncorrelation. Hansen’s over identification test shows that our instrument variable is valid. Taken together, our construction of instrument variable is reliable and effective. The results of Model 1 and Model 3 show that there is a significant and negative relationship between market segmentation and energy efficiency, which is consistent with the conclusions from existing studies [13, 14]. In Model 2 and Model 4, the coefficients of *l*.*lnEE* are positive and statistically significant, suggesting that energy efficiency is not only affected by present factors, but also positively related to past energy efficiency. More importantly, these results document the inverse U-shaped relationship between market segmentation and energy efficiency, with coefficient of *lnMS* and (*lnMS*)^2^ being negative and significant at conventional levels. Therefore, our main findings are valid and robust.

#### Threshold regression

To confirm the non-linear relationship between market segmentation and energy efficiency, we estimated the following threshold regression modelling:

$$\text{ln}EE=\delta_{0}+\delta_{1}\text{ln}MS_{it}I(\text{ln}MS_{it}<\varphi )+\delta_{2}\text{ln}MS_{it}I(\text{ln}MS_{it}>\varphi )+\theta X_{it}+\mu_{i}+\varepsilon_{it}$$ (10)

where ln*MS* is the threshold variable and *φ* is the threshold value; *I*(·) is an indicator function, the value of *I*(·) equals 1 when its argument is true, otherwise, equals 0; so *δ*_1_ and *δ*_2_ reflect marginal effect.

**Fig 4:**
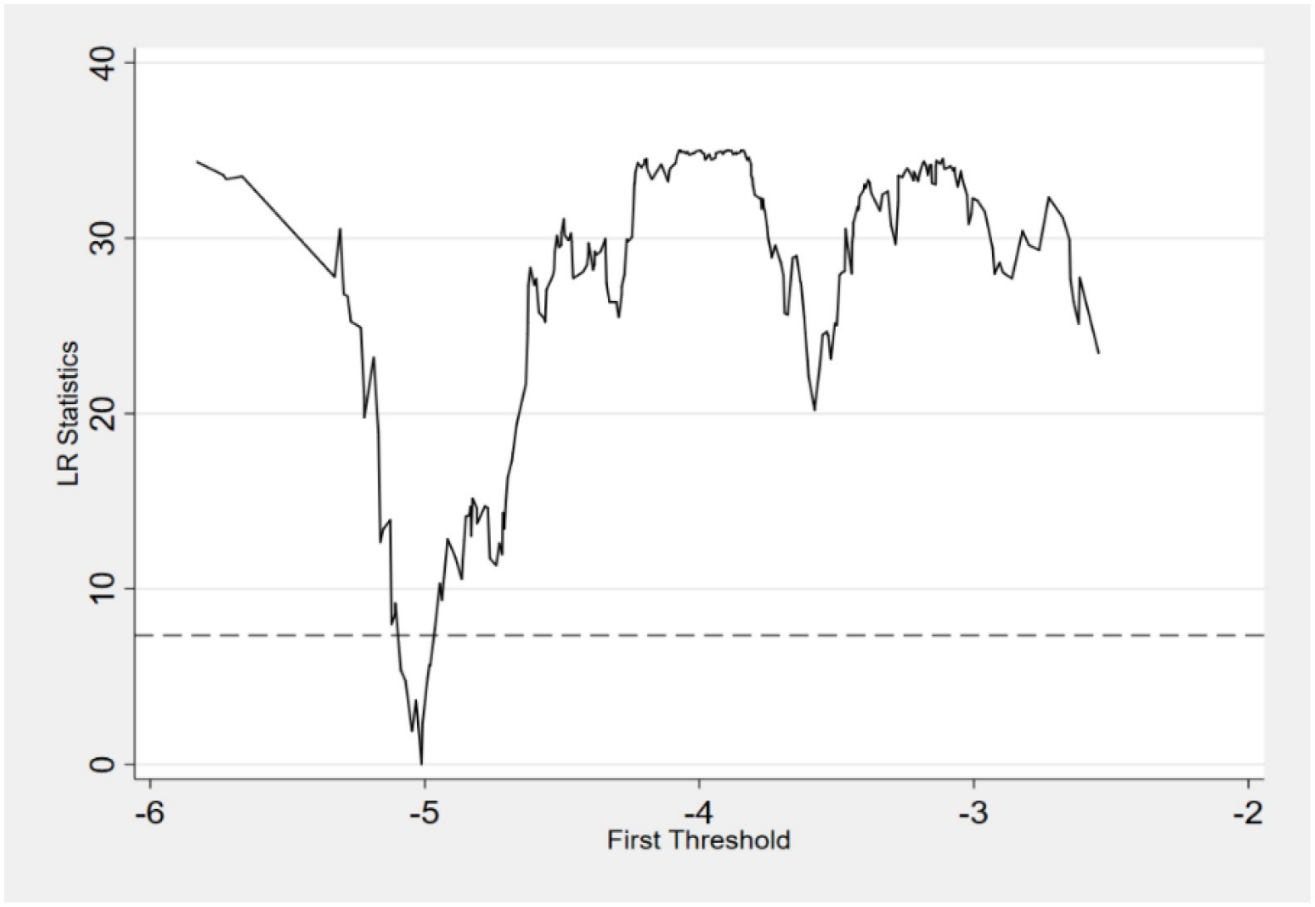
LR statistics of threshold regression.

As a priliminary step of threshold regression, we implemented single threshold test and double threshold test to select the number of cut-off points. As showed in Table 6, the value of Threshold 1 is -5.0114 with the F statistic at a 1% significant level, implying a rejection for the linear relationship between market segmentation and energy efficiency at 1% significance level. In other words, there exists at least one changing point in the curve. The results of Threshold 2 suggests that we cannot reject the null hypothesis that there are less than 2 cut-off points. At the same time, Fig 4 is the likelihood ratio function chart drawn according to the single threshold model. With the help of the likelihood ratio function chart, the threshold estimation value and confidence interval can be observed more intuitively. Taken together, we use single threshold for panel threshold regression with fixed effects.

**Table 6:** Threshold effect test results.

Model	Threshold value	F statistic	P-value
**Threshold 1**	-5.0114	67.54	0.0000
**Threshold 2**	-5.4572	12.74	0.1140

Table 7 shows the main results of the threshold regression modelling. It is clear that the coefficient of market segmentation is negative and statistically significant when the MS index is greater than the threshold (-5.0114), implying a detrimental impact of market segmentation on energy efficiency. In constrast, a positive and significant influence of market segmentation on energy efficiency is observed when the MS index is less than the threshold value. The results further confirm that our main findings are robust and reliable.

**Table 7:** Threshold regression results.

	Threshold
***LnMS(LnMS<*-5.0114)**	0.0028*
	(0.0016)
***LnMS(LnMS>*-5.0114)**	-0.0031*
	(0.0018)
***Intercept***	-1.7219
	(1.3506)
***Control variables***	YES
***withR***^***2***^	0.1644
***N***	540

*** p<0.01,

** p<0.05,

* p<0.1.

## Conclusion

Prior literatures tend to propose and examine the negative relationship between market segmentation and energy efficiency. However, the picture of the segmentation-efficiency nexus is distorted if the role of local government is ignored. Therefore, with a consideration for China’s specific characteristics, we propose a non-linear relationship between market segmentation and energy efficiency. Using data of 30 provinces in Mainland China during 2000 to 2017, we find an inverse U-shaped relationship between market segmentation and energy efficiency. Specifically, when market segmentation is low, energy efficiency increases as market segmentation increases; however, when market segmentation approaches a certain high level, the positive impact disapears and becomes negative. Therefore, either too low or too high level of market segmentation is not conducive to improving energy efficiency. Rather, a moderate extent of market segmentation is beneficial for improvement of energy efficiency. To confirm empirical findings, we utilise GMM and threshold regression modelling to examine its robustness. Our main findings are robust after controlling for endogeneity problems. Notably, in contrast to existing literatures using DEA and traditional SFA methods to measure energy efficiency, this research makes use of the recently developed SFA model to measure energy efficiency, which not only takes into account the unobserved heterogeneity, but also captures information on individual heterogeneity, time-variant inefficiency, time-invarient inefficiency, and random shocks. The use of this method thus improves measurement accuracy of energy efficiency.

Our findings tell a more nuanced and complete story about the relationship between market segmentation and energy efficiency. We show that market segmentation does not necessarily lead to a decrease in energy efficiency; moderate level of market segmentation is optimal for improving energy efficiency in China. Our findings provide important policy implications. Firstly, the central government should keep market segmentation within a certain range. In some areas with high degrees of market segmentation, the central government should promote the process of marketisation, and use legal measures to restrict regional market segmentation. In regions with low levels of market segmentation, the central government should encourage local governments to leverage appropriate tools to protect local firms and products in order to increase market segmentation to a moderate level. Secondly, we suggest that the central government should increase the assessment of the local environmental assessment and encourage local governments to promote inter-regional trade openness and healthy competition environment to improve energy efficiency. Thirdly, in order to strengthen the regualtion of energy conservation and emission reduction, the central government could set up a regional energy conservation and emission reduction supervision agency that is independent of local governments. Finally, entreprises should establish strategic plans for long-term development, make better use of various protection and preferential policies, and actively invest in advanced production technologies to continuously improve their core competitiveness.

Importantly, although our findings are based solely on Chinese sample, the findings are generalisable to emerging economies because: 1) China is a typical emerging economy with imperfect market mechanisms and significant interventions from central and local governments; 2) China is the world’s second-largest economy and the largest energy consumer. The inverse U-shaped relationship between market segmentation and energy efficiency is thus applicable and meaningful for other emerging economies.

## Supporting information

S1 Data (RAR)
